# Psychological safety as a context-sensitive predictor of retention intentions: Gendered effects of supervisor support under caregiving-assumed conditions

**DOI:** 10.1371/journal.pone.0346791

**Published:** 2026-04-06

**Authors:** Michi Yoshimura, Hiroyuki Yamaguchi

**Affiliations:** 1 Faculty of Business Administration, Aichi Toho University, Nagoya, Japan; 2 Faculty of Comprehensive Psychology, Kyoto Tachibana University, Kyoto, Japan; Pingdingshan University, CHINA

## Abstract

This study examined how emotional and instrumental supervisor support influence employees’ retention intentions through psychological safety, with a particular focus on gender and caregiving contexts. An online questionnaire survey was conducted among 522 Japanese employees (248 men and 274 women), and the hypothesized model was tested using structural equation modeling (SEM), multi-group invariance analysis, and bootstrapping. The analysis revealed clear differences in the indirect effects of emotional support on retention intentions via psychological safety between the general working condition and the caregiving-assumed condition. Under the general condition, the indirect effects were statistically significant for both men and women; however, the standardized indirect effects (*β*) were small in magnitude, particularly among men. In contrast, when employees assumed future caregiving responsibilities, the magnitude of the mediated pathway increased substantially, and the influence transmitted through psychological safety became markedly stronger. Furthermore, the direct effect of psychological safety on retention intentions also exhibited context-dependent variation. Among men, this direct path was non-significant under the general condition but became significantly and markedly stronger under the caregiving-assumed condition. Among women, the direct effect was significant under both conditions but was notably stronger when caregiving responsibilities were assumed. In contrast, instrumental support did not show significant effects under either condition, suggesting that practical assistance alone may be insufficient to enhance psychological safety or retention intentions when interpersonal risk is salient. Taken together, these findings suggest that when employees anticipate future caregiving burdens, the strength of the influence transmitted through psychological safety increases, indicating that the effectiveness of emotional support varies considerably depending on contextual demands. These findings highlight the importance of psychological safety as a context-sensitive mechanism linking supervisor support to retention intentions, particularly under anticipated caregiving conditions.

## 1. Introduction

This study aims to examine how family caregiving burdens—such as childcare and eldercare—may undermine employees’ psychological safety and heighten concerns about continued employment, and how these effects are compensated for by supervisors’ emotional and instrumental support, with particular attention to gender differences. In this context, when family caregiving increases interpersonal risk and anxiety, supervisor support functions as an important resource that sustains employees’ psychological and behavioral outcomes. Many studies have consistently demonstrated that supervisor support plays a critical role in sustaining employees’ work engagement and continued employment. High-quality relationships between supervisors and subordinates have been shown to enhance job satisfaction and organizational commitment [5]. Research on Perceived Organizational Support (POS) also indicates that supervisors function as agents of the organization and strengthen employees’ sense of organizational belonging [8,9]. Furthermore, supervisor support has been reported to buffer the detrimental effects of role stressors and to enhance subordinates’ job satisfaction and involvement [1]. In Japan, studies of female workers have shown that emotional support from supervisors contributes to reducing psychological stress [19]. Taken together, these findings suggest that supervisor support serves as a broad workplace resource that sustains employees’ psychological and behavioral outcomes.

At the same time, theoretical frameworks for understanding social support emphasize its qualitative dimensions. Support is not a unitary resource but can be categorized into multiple types, such as emotional support (e.g., empathy, affirmation) and instrumental support (e.g., task coordination, problem solving) [14]. Recent meta-analytic evidence on Family-Supportive Supervisor Behaviors (FSSB) indicates that supervisor support can operate through multiple psychological mechanisms [12]. Evidence from this meta-analysis demonstrates that resource-based pathways primarily account for FSSB’s effects on well-being outcomes such as burnout, whereas social-exchange-based pathways more strongly explain job-related outcomes such as satisfaction and performance [12]. Building on these insights, the present study assumes that instrumental support is more likely to activate resource‐based processes, whereas emotional support is more closely tied to social‐exchange processes.

A psychological mechanism that has attracted increasing attention as a link between supervisor support and continued employment is psychological safety. Initially defined as a state in which individuals can express themselves without fear of negative consequences [17], psychological safety was later conceptualized as a shared belief that the team is safe for interpersonal risk taking [6]. Psychological safety has been shown to relate to a wide range of positive outcomes, including engagement and commitment [27]. Evidence from prior research further shows that the three psychological conditions—meaningfulness, safety, and availability—enhance employee engagement [23]. This work also indicates that supportive relationships with supervisors play an important role in fostering psychological safety [23]. These findings align with previous research showing that high-quality interpersonal relationships in the workplace support psychological safety and, in turn, promote employees’ positive outcomes [3]. Moreover, a large-scale meta-analysis demonstrated that psychological safety is consistently positively associated with task performance and organizational citizenship behavior, and that supportive leadership behaviors function as key antecedents of psychological safety [10]. Although the importance of psychological safety is relatively stable under typical workplace conditions, its relevance becomes even more pronounced in situations where interpersonal risk is elevated. In Japan, institutional and cultural factors—such as long working hours and traditional gender-role divisions—continue to hinder sustained employment, and multiple studies have shown that these structural constraints exacerbate work–family conflict [16,22,24,26,32]. Such structural constraints have also been observed internationally, with gender differences in career continuity remaining prevalent [11]. Although these issues are particularly salient in Japan, similar patterns of caregiving-related work tension have been documented internationally, indicating that these mechanisms are not culturally specific. Family caregiving responsibilities may contribute to psychological stress due to the unpredictable nature of childcare and eldercare demands [13], generate conflicts between ideal and actual self-images in the workplace [15], and may even induce feelings of guilt or social exclusion when receiving workplace accommodations [24].

The significance of psychological safety lies in its function beyond merely fostering a sense of being supported. While emotional support provides reassurance and instrumental support facilitates task execution, psychological safety operates as a foundational resource that enables employees to disclose constraints, express needs, and seek necessary adjustments. In this sense, psychological safety is a higher-order psychological resource that reduces interpersonal risk and enables open, candid interactions in the workplace.

Under such conditions, emotional support may strengthen reassurance and thereby enhance psychological safety, whereas instrumental support—while useful for task execution—may have limited influence on reducing interpersonal risk directly. Moreover, the extent to which psychological safety relates to intentions to continue working is likely to vary depending on whether employees anticipate assuming caregiving responsibilities.

Therefore, it is important to examine how different types of supervisor support (emotional vs. instrumental) influence employees’ intentions to remain employed through psychological safety, particularly under conditions in which caregiving responsibilities are anticipated.

Gender differences were examined in this study because anticipated caregiving responsibilities may carry fundamentally different psychological implications for men and women. In particular, childbirth and early childcare impose biological and social demands that are experienced exclusively or disproportionately by women, making caregiving-related career decisions more personally salient and psychologically consequential. Even before caregiving responsibilities arise, women may anticipate potential career disruptions and work–family conflicts, reflecting gendered career expectations shaped by broader social and structural contexts. These anticipatory considerations can influence career planning, employment continuity decisions, and perceptions of long-term career sustainability.

In such contexts, the supervisor plays a critical role as the most immediate organizational representative shaping employees’ perceptions of support and acceptance. Psychological safety fostered through supportive supervisor relationships may therefore serve as a key psychological mechanism influencing whether employees perceive continued employment as viable under anticipated caregiving demands. Examining gender differences in this process enables a more precise understanding of how anticipatory caregiving concerns interact with relational workplace factors to influence retention intentions.

More specifically, despite these theoretical implications, few studies have systematically examined how emotional and instrumental support differentially influence retention-related processes in caregiving-assumed contexts, or how these processes may vary by gender. To address this gap, the present study investigates how supervisors’ emotional and instrumental support influence employees’ intentions to remain employed through psychological safety, focusing on

(1) how this mediating process differs between a general condition and a caregiving-assumed condition, and
(2) the extent to which these processes vary between men and women.

**Hypothesis 1 (Emotional Support – Mediated Effect)**

Emotional support will positively influence retention intentions through psychological safety. This indirect effect will be stronger under the caregiving-assumed condition and may vary in magnitude between men and women.

**Hypothesis 2 (Instrumental Support – Limited or Contextual Effect)**

Instrumental support will show limited or no indirect effect on retention intentions through psychological safety. Its impact, if present, may depend on gender or caregiving assumptions.

**Hypothesis 3 (Psychological Safety – Contextual Strengthening)**

Psychological safety will have a stronger positive effect on retention intentions when employees anticipate family caregiving responsibilities, with the potential for gender differences in standardized direct effect (*β*).

## 2. Methods

### 2.1 Ethics statement

This study was approved by the Ethics Review Committee of Aichi Toho University (Approval No. 202304). All participants were provided with a written explanation of the study aims and data-handling procedures and gave informed consent prior to participation. The study was conducted in accordance with the institutional ethical guidelines.

### 2.2 Survey participants and procedures

This study employed a cross-sectional survey design. Data were collected on March 24, 2024 through an online research company using a registered panel of participants. Eligible participants were full-time employees in their 20s or 30s residing in the Kanto region of Japan (Tokyo, Kanagawa, Chiba, Saitama, and Ibaraki) and without children.

Participants were informed about the study purpose, voluntary participation, confidentiality, and data handling procedures, and provided electronic informed consent by checking an agreement box before accessing the questionnaire. Initially, 600 responses were collected with an equal number of male (*n* = 300) and female (*n* = 300) participants. All participants were adults aged 20 years or older.

This study focused on participants without children to ensure that the caregiving-assumed condition functioned as a hypothetical, future-oriented scenario rather than reflecting ongoing caregiving responsibilities. Including individuals with active caregiving duties could introduce confounding influences from their real experiences and coping strategies, thereby obscuring the psychological meaning of the assumed caregiving condition. Restricting the sample in this way enhanced the internal validity of the manipulation. After excluding 78 cases (52 men [17.3%] and 26 women [8.7%]) due to uniform response patterns (i.e., straight-lining), the final dataset comprised 522 participants (248 men and 274 women; mean age = 30.34 years, *SD* = 5.15). The participants received a predetermined number of points from the research company as compensation. Structural equation modeling (SEM), multi-group invariance testing, and bootstrapped indirect effect analyses were performed to examine the effects of supervisor support. Analyses were conducted using SPSS version 26.0 and AMOS version 30.0 (IBM Corp.).

Structural equation modeling (SEM) was employed because the primary objective of this study was to examine theoretically derived structural relationships among latent psychological constructs, including emotional support, instrumental support, psychological safety, and retention intentions. SEM allows simultaneous estimation of measurement and structural components while accounting for measurement error, providing a rigorous framework for testing mediation mechanisms and comparing structural relationships across different contextual conditions and gender groups. This approach was particularly appropriate for evaluating the hypothesized mediating role of psychological safety and its context-dependent effects.

### 2.3 Measurement scales and constructs

All multi-item scales used in this study were originally developed in Japanese or formally translated and validated by Japanese researchers, with their reliability and construct validity confirmed in prior studies involving Japanese working populations.

#### 2.3.1 Retention intentions.

Participants’ intentions to continue working were assessed using two single-item measures:

(1) intention to remain employed under general conditions and
(2) intention to remain employed under assumed family-care conditions.

Parallel items were developed to ensure conceptual consistency across the two conditions. Participants responded using a 5-point Likert scale (1 = strongly disagree, 5 = strongly agree). In the present study, single-item measures were used to assess retention intention under caregiving-assumed and general conditions, allowing direct comparison of the same behavioral intention across conditions. Prior research has shown that when the construct being measured is sufficiently specific and unambiguous to respondents, single-item measures can function effectively [29].

For example, substantial correlations (*r* = .63–.72) between single-item and multi-item measures of overall job satisfaction have been documented, demonstrating the empirical validity of the single-item approach [31]. They also estimated potential reliability coefficients ranging from.45 to.69 using the correction for attenuation formula, suggesting acceptable stability. Similarly, analyses demonstrated comparable correlations (*r* = .60–.72) at the facet level of job satisfaction, reaffirming that when the construct is conceptually clear, single-item measures can be both valid and practically useful [25].

#### 2.3.2 Emotional and instrumental support.

Supervisor support was measured using a social support scale that was originally developed and later revised and expanded in subsequent work [18,19]. Emotional support was assessed with eight items (e.g., “He/she encourages me when I am feeling down at work”) and instrumental support with six items (e.g., “He/she provides me with knowledge and information that I can apply to my work”). Responses were recorded on a five-point Likert scale, with higher scores indicating stronger perceived support. Exploratory factor analyses (maximum likelihood method with Promax rotation) were conducted separately for each subscale, confirming a one-factor solution for both emotional and instrumental support, with all factor loadings exceeding.70. The emotional and instrumental support scales demonstrated high internal consistency (Cronbach’s α = .92 and.91, respectively).

#### 2.3.3 Psychological safety.

Psychological safety was measured using the Japanese version of the scale in [28], adapted and validated in [30]. This scale includes separate subscales for leader and coworker psychological safety. In the present study, only the leader psychological safety subscale was used, as it was theoretically aligned with the focus on supervisor support. The scale consists of nine items rated on a seven-point Likert scale (e.g., “When I express my opinion, the team leader [or supervisor] listens to it with respect”). Higher scores indicated a greater sense of psychological safety. An exploratory factor analysis (maximum likelihood method with Promax rotation) supported a one-factor solution for this construct, with all factor loadings exceeding.70. Note that supervisor support was rated on a five-point scale, whereas psychological safety was rated on a seven-point scale. The psychological safety scale also demonstrated high internal consistency in the present sample (Cronbach’s α = .93).

### 2.4 Parceling strategy

Following the recommended item-to-construct balance procedure [21], items were assigned to three parcels such that the average factor loadings were approximately equivalent across parcels. This parceling approach is considered appropriate when constructs exhibit clear unidimensionality, as it can improve indicator reliability and stabilize parameter estimation in structural equation modeling [21].

In the present study, factor analyses confirmed that the scales for emotional support, instrumental support, and psychological safety were unidimensional and theoretically coherent. Therefore, items with relatively higher and lower factor loadings were distributed evenly across parcels to ensure that each parcel adequately represented the underlying construct and contributed equally to parameter estimation in the structural equation models.

## 3. Results

### 3.1 Preliminary analyses

Multicollinearity was assessed using variance inflation factors (VIFs) under both the general and caregiving-assumed conditions. All VIF values were below 3.1, indicating no concern of multicollinearity. Table 1 presents the means, standard deviations, internal consistencies, and correlations among the study variables (*n* = 522). All scales demonstrated excellent internal reliability (Cronbach’s *α* > .90), and exploratory factor analyses confirmed one-factor solutions for each construct (see S1 Table for details). All variables showed acceptable normality, with skewness and kurtosis values within the recommended range (±2.0). Emotional and instrumental support were highly correlated with each other, and both were positively associated with psychological safety and intentions to continue working (see Table 1).

**Table 1:** Descriptive statistics and correlations among variables (*N* = 522; Men = 248, Women = 274).

Variable		*M*	*SD*	*α*	1	2	3	4
1	Emotional Support	3.10	0.91	.92	—			
2	Instrumental Support	3.13	0.93	.91	.79***	—		
3	Psychological Safety	4.20	1.18	.93	.60***	.52***	—	
4	Retention Intention (general)	2.96	1.20	—	.43***	.42***	.36***	—
5	Retention Intention (caregiving-assumed)	3.86	1.57	—	.44***	.42***	.56***	.61***

**Note:** All correlations are significant at *p* < .001. *M* = mean; *SD* = standard deviation; *α* = Cronbach’s alpha.

* *p* < .05, ** *p* < .01, *** *p* < .001.

Correlations among parcels and outcome variables are presented in S1 Table.

### 3.2 Structural equation modeling

**Fig 1:**
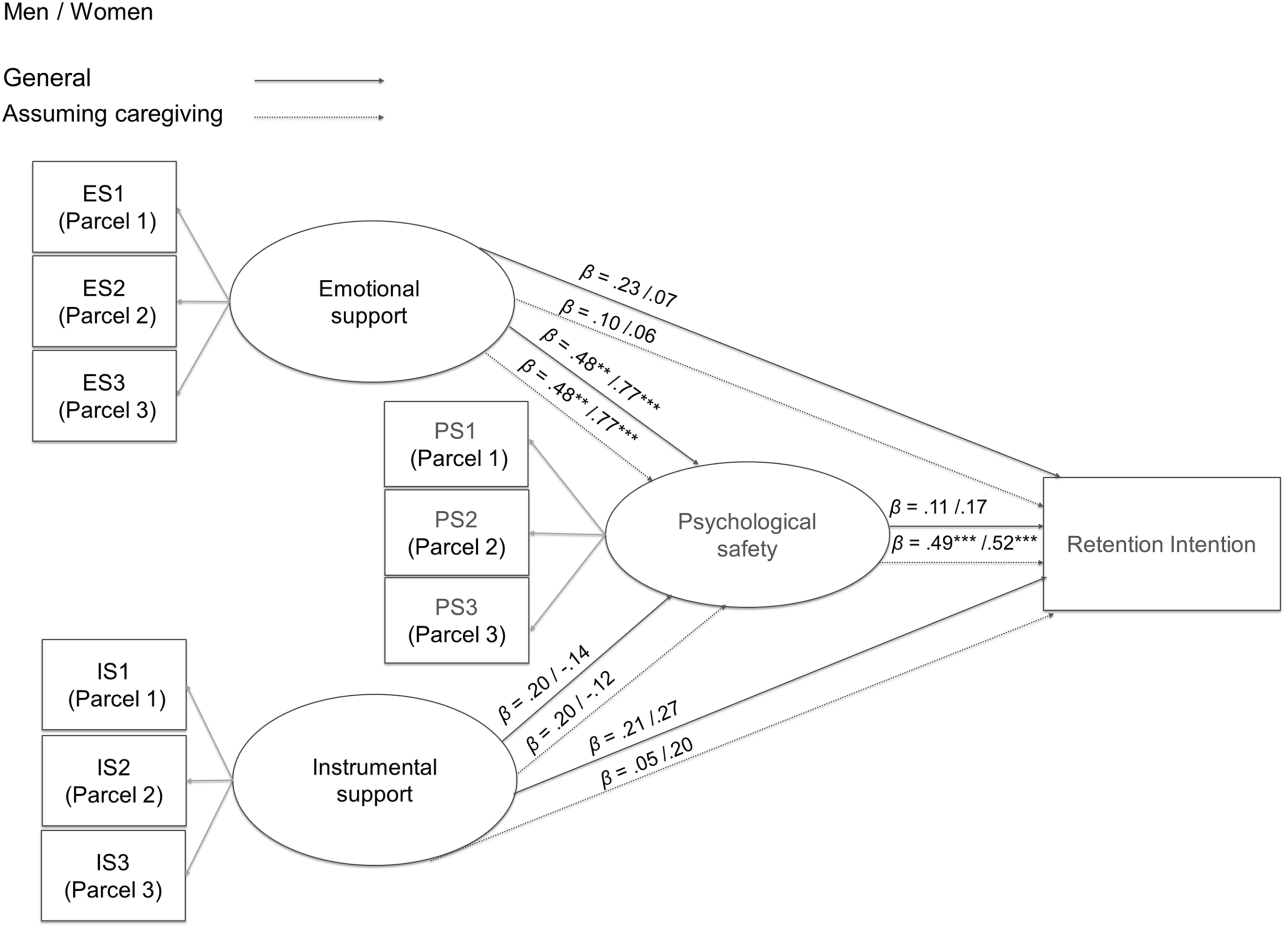
Structural equation models across gender and caregiving-assumed conditions. **Note.** Solid lines represent the general condition, and dotted lines represent the caregiving-assumed condition. Standardized path coefficients (*β*) are shown, with male values on the left and female values on the right of each path. Each latent construct was represented by three parcels (e.g., ES1–ES3) created using the balancing method [21]. * *p* < .05, ** *p* < .01, *** *p* < .001.

Having confirmed satisfactory reliability and validity of the measurement model, we next tested the hypothesized structural relationships using structural equation modeling (SEM). As shown in Fig 1, the hypothesized model included emotional and instrumental supervisor support, psychological safety, and retention intentions. To reflect the theoretical assumptions outlined in the introduction, the structural model specified emotional and instrumental supervisor support as predictors of psychological safety, which was modeled as the central mediating mechanism linking supervisor support to employees’ retention intentions, particularly under caregiving-assumed conditions.

Fig 1 illustrates the standardized structural paths for males and females under both the general and caregiving-assumed conditions. Model fit was evaluated using conventional indices, including the chi-square statistic (*χ²*), comparative fit index (CFI), Tucker–Lewis index (TLI), root mean square error of approximation (RMSEA), and standardized root mean square residual (SRMR).

Two models were tested: (1) the general condition and (2) the caregiving-assumed condition. Model fit indices indicated good to excellent fit across both models, confirming that the hypothesized structure adequately represented the data (see Table 2).

**Table 2:** Model fit indices for SEM and multi-group invariance tests (*N* = 522; Men = 248, Women = 274).

Model	*χ² (df)*	CFI	TLI	RMSEA (CI)	SRMR	Δ*χ²(df*)	ΔCFI	ΔRMSEA
SEM (General)	108.28(30)	.982	.972	.071 [.057,.085]	.028	—	—	—
SEM (Caregiving)	134.65(30)	.976	.964	.082 [.068,.096]	.029	—	—	—
**General**								
*Configural invariance*	150.65(60)	.979	.968	.054 [.045,.065]	.030	—	—	—
*Metric invariance*	151.93(66)	.980	.972	.050 [.040,.061]	.025	1.28 (6)	.001	−.004
*Scalar invariance*	169.80(76)	.978	.974	.049 [.039,.059]	—	17.79 (10)	−.002	−.001
**Caregiving-assumed**								
*Configural invariance*	181.44(60)	.972	.958	.062 [.052,.073]	.036	—	—	—
*Metric invariance*	183.31(66)	.973	.963	.058 [.049,.069]	.026	1.88 (6)	.001	−.004
*Scalar invariance*	201.09(76)	.971	.966	.056 [.047,.066]	—	17.78 (10)	−.002	−.002

**Note:** Measurement invariance was evaluated using ΔCFI ≤ .010 [4]. All models showed acceptable to excellent fit across gender groups. SRMR was not reported for scalar invariance models because AMOS does not provide SRMR estimates for models with equality constraints across groups. *χ²* = chi-square; *df* = degrees of freedom; CFI = comparative fit index; TLI = Tucker–Lewis index; RMSEA (90% CI) = root mean square error of approximation; SRMR = standardized root mean square residual.

These results suggest that the hypothesized structure was appropriate for subsequent multi-group analysis.

### 3.3 Multi-group invariance analysis by gender

To examine gender differences, multi-group analyses were conducted at three levels of invariance: configural, metric, and scalar models. Measurement invariance tests were performed for both the general and caregiving-assumed conditions. Across all levels of invariance, the models demonstrated acceptable fit indices, with ΔCFI and ΔRMSEA values remaining within the recommended thresholds. Results of the multi-group invariance tests are summarized in Table 2. Overall, the analyses supported metric and scalar invariance, indicating that the latent structure was comparable between men and women.

In addition, indirect effects were estimated using the bootstrap method. The detailed standardized coefficients for direct and indirect effects, estimated through bootstrapping, are presented in Table 3.

**Table 3:** Direct and indirect effects by gender (*N* = 522; Men = 248, Women = 274).

Path	Group	*β* (Std.)	95% CI (BC)
**General**			
Emotional → Psych. Safety → Retention	Men	.05	[.01,.16]
Emotional → Psych. Safety → Retention	Women	.13	[.02,.26]
Instrumental → Psych. Safety → Retention	Men	.02	[−.01,.10]
Instrumental → Psych. Safety → Retention	Women	−.02	[−.09,.01]
**Caregiving-assumed**			
Emotional → Psych. Safety → Retention	Men	.24	[.09,.44]
Emotional → Psych. Safety → Retention	Women	.40	[.27,.57]
Instrumental → Psych. Safety → Retention	Men	.10	[−.05,.25]
Instrumental → Psych. Safety → Retention	Women	−.07	[−.19,.06]
Psych. Safety → Retention (direct)	Men	.49	[.33,.63]
Psych. Safety → Retention (direct)	Women	.51	[.39,.63]

**Note:** All coefficients are standardized (*β*). Confidence intervals are bias-corrected (BC) based on 5,000 bootstrap resamples. Indirect paths represent mediation via psychological safety. Separate models were estimated for the general and caregiving-assumed conditions. Direct effects from emotional and instrumental support to retention intentions were nonsignificant across all conditions. Therefore, the indirect effects through psychological safety are presented as the primary indicators of mediation.

### 3.4 Indirect effects

The indirect effects were examined using bias-corrected bootstrapping (5,000 resamples).

Under the caregiving-assumed condition, emotional support exerted a significant indirect effect on retention intentions through psychological safety for both men (*β* = .24) and women (*β* = .40).

Under the general condition, the indirect effects were statistically significant for both men and women; however, their magnitudes were clearly weaker than those observed under the caregiving-assumed condition, indicating that the influence mediated by psychological safety was more limited in this context (men *β* = .05, *CI* [.01,.16]; women *β* = .13, *CI* [.02,.26]).

These results indicate that emotional support appears to enhance employees’ retention intentions largely through increased psychological safety when caregiving responsibilities are assumed.

In contrast, the indirect effects of instrumental support through psychological safety were nonsignificant across both genders and conditions, suggesting that instrumental assistance from supervisors has a consistently limited influence on employees’ sense of security and intention to remain.

Taken together, these findings demonstrate that psychological safety functions as a key mediating mechanism linking emotional support to retention intentions, particularly in contexts where caregiving is cognitively salient (see Table 3).

## 4. Discussion

This study examined how different forms of supervisor support—emotional and instrumental—affect employees’ intentions to remain in the workforce through psychological safety, taking into account gender and caregiving contexts. By comparing a general condition and a caregiving-assumed condition, this research provides empirical insight into how the meaning and stability of supervisor support change when family responsibilities are cognitively activated.

The study extends prior work on supervisor support and turnover intentions by conceptualizing psychological safety as a cross-domain psychological resource—one that functions not merely as a workplace climate variable, but as a relational buffer that connects professional and family-role expectations.

### 4.1 Emotional support

Consistent with Hypothesis 1, emotional support positively influenced retention intentions through psychological safety, and this mediating relationship became more pronounced when caregiving responsibilities were assumed. The magnitude of this pathway increased substantially under the caregiving-assumed condition and varied in strength between men and women, suggesting that gendered norms surrounding work and family roles may shape how relational support is perceived. These findings indicate that emotional support from supervisors serves as a primary source of psychological safety, and becomes particularly indispensable for sustaining employees’ continued employment in contexts where interpersonal risks associated with future caregiving demands are heightened.

Moreover, in Japanese workplaces, employees—especially women—often experience psychological tension when balancing work and caregiving, driven by concerns about burdening colleagues or being perceived as less committed to their jobs. Such interpersonal concerns likely amplify the value of psychological safety as a relational resource, helping employees communicate their constraints without fear of negative evaluation.

Taken together, these results highlight that psychological safety functions as a relational resource whose importance increases depending on contextual demands. Although it plays a role under general conditions, its relevance becomes especially salient in situations where anticipated caregiving responsibilities heighten concerns related to interpersonal risk.

### 4.2 Instrumental support

In this study, instrumental support showed neither direct nor indirect associations with retention intentions across any gender or condition, indicating that Hypothesis 2, which predicted limited or absent effects, was supported.

Although instrumental assistance—such as task help, schedule flexibility, or informational guidance—is theoretically expected to facilitate work–life balance, its effects were consistently weak and did not differ by gender or caregiving context. The absence of significant effects suggests that employees may perceive instrumental support as situational or transactional, lacking the relational depth necessary to generate psychological safety. In this study, instrumental support did not significantly predict psychological safety for either men or women, reinforcing the interpretation that practical help alone does not cultivate the interpersonal trust required for sustained employment commitment.

One possible explanation for the nonsignificant effects of instrumental support is that instrumental assistance primarily addresses task-related demands rather than interpersonal risk, which is central to psychological safety. Psychological safety reflects employees’ perceptions that they can express concerns or vulnerabilities without fear of negative interpersonal consequences, a perception fundamentally grounded in relational trust rather than logistical assistance [6].

While instrumental support may help employees manage workload or logistical challenges, it may not directly influence employees’ perceptions of being accepted, understood, or psychologically secure in expressing potential work–family conflicts. Thus, instrumental support may improve functional capacity without necessarily strengthening the relational assurance required to sustain long-term employment intentions.

Furthermore, because the present study examined anticipated caregiving situations rather than current caregiving experiences, participants’ evaluations likely reflected psychological expectations about future relational support rather than immediate practical needs. In such anticipatory contexts, emotional support may play a more salient role in shaping employees’ perceptions of whether continued employment is psychologically feasible, whereas instrumental support may be perceived as conditional or situation-specific. This distinction helps explain why emotional support, but not instrumental support, was significantly associated with psychological safety and retention intentions.

Taken together, these findings highlight the importance of distinguishing between practical accommodations and relational support when evaluating supervisor behaviors. This study suggests that although instrumental support may alleviate immediate work demands, it is insufficient to promote long-term retention through psychological safety, particularly when employees evaluate their future employment in the context of anticipated caregiving responsibilities.

### 4.3 Psychological safety

The present study demonstrated that the effect of psychological safety on retention intentions varies substantially depending on the context. Under the general condition, psychological safety showed a small positive effect only for women, whereas no significant effect was observed for men. This pattern likely reflects gendered dynamics in Japanese workplaces, where women tend to be more attuned to interpersonal evaluations and concerns about imposing burdens on colleagues, making relational resources more central to their decisions about continued employment. Men, in contrast, may rely more heavily on structural or extrinsic factors—such as income stability or promotion prospects—leading psychological safety to play a less direct role in their retention decisions under routine circumstances.

However, when caregiving responsibilities were assumed, the pattern shifted markedly. Anticipated caregiving demands heighten interpersonal risks for both genders, including potential increases in absenteeism, the need for workload adjustments, concerns about burdening coworkers, and fears of being perceived as less committed. Psychological safety tends to be less accessible to individuals in weaker or more vulnerable positions, such as those with lower social status or limited career experience [7]. In such contexts, psychological safety emerged as a critical determinant of retention intentions for both men and women, showing strong and nearly identical positive effects (*β* = .49–.51), indicating a substantial increase compared to the general condition.

Taken together, these findings indicate that psychological safety is not a gender-specific predictor of retention but a context-sensitive psychological resource whose importance increases when interpersonal risks are salient. When caregiving responsibilities become cognitively activated, psychological safety functions as a universally meaningful mechanism that supports continued employment across individuals, rather than as a factor relevant only to specific groups.

This context-dependent pattern can be understood in light of theoretical frameworks emphasizing the anticipatory nature of major life and career decisions. According to the theory of planned behavior, behavioral intentions are formed through individuals’ evaluations of expected outcomes, perceived social expectations, and perceived feasibility [2]. In the context of family and career planning, fertility intentions and related life decisions are shaped not only by current circumstances but also by perceived constraints and expectations regarding future life courses. Empirical evidence further indicates that young adults’ family size intentions are adjusted over time in response to changes in partner, educational, and occupational careers, as well as the timing of childbearing [20]. Taken together, these perspectives suggest that examining retention intentions under an anticipated caregiving condition provides a theoretically grounded approach for understanding how psychological safety and supervisor support may influence employees’ longer-term career sustainability.

## 5. Theoretical and practical implications

The results of this study revealed that, under the general condition, the influence of supervisor support on employees’ retention intentions was generally limited. Although the indirect effect of emotional support through psychological safety reached significance for women, its magnitude was small, indicating that the practical impact of this pathway may be modest.

In contrast, under the caregiving-assumed condition, emotional support demonstrated clear and robust indirect effects for both men and women, indicating a substantial strengthening of the mediating role of psychological safety.

These findings suggest that psychological safety is shaped not merely by instrumental forms of support, such as task-related assistance or scheduling flexibility, but also by employees’ emotional experience of being understood, respected, and supported by their supervisors.

In this sense, psychological safety can be understood as a relational psychological resource that emerges from interpersonal trust and emotional reassurance. Its role appears to become more pronounced in situations involving uncertainty or anticipated role conflict, such as balancing work and caregiving responsibilities.

This finding contributes to the psychological safety literature by highlighting the contextual conditions under which emotional support becomes more strongly associated with employee retention intentions.

From a practical standpoint, the results highlight the importance of distinguishing emotional from instrumental support in supervisory practice. Consequently, supervisor training programs should incorporate not only task-related support skills but also relational competencies such as empathy, active listening, and providing reassurance.

Furthermore, organizations should develop mechanisms—such as regular check-ins, structured procedures for workload negotiation, and confidential consultation services—that allow employees to safely disclose concerns about future caregiving demands.

Such organizational practices can reduce the stigma associated with discussing work–family conflict and may ultimately enhance employees’ retention intentions, particularly among those who anticipate future caregiving responsibilities.

## 6. Limitations and future research

Nevertheless, several limitations should be noted. While this study provides valuable insights into how supervisor support and psychological safety interact to influence retention intentions, several limitations warrant consideration.

First, the cross-sectional design precludes causal inference, and future longitudinal or experimental studies are needed to examine the temporal sequence among emotional support, psychological safety, and retention outcomes.

Second, retention intentions were assessed using single-item measures. Although prior research supports the validity of single-item measures for clearly defined constructs, such measures may not fully capture the multidimensional nature of retention intention. The use of single-item measures in this study was intended to ensure conceptual equivalence and minimize respondent burden when comparing retention intentions across hypothetical and general conditions. Future research using multi-item scales would help to further strengthen measurement reliability and provide a more comprehensive assessment.

Third, caregiving was assessed as a hypothetical or anticipated condition rather than an ongoing reality, which may limit ecological validity because hypothetical scenarios cannot fully capture the psychological and practical demands of actual caregiving experiences. However, this approach is theoretically justified, as career-related intentions and decisions are shaped not only by current experiences but also by individuals’ anticipatory evaluations of future life-course roles, including caregiving responsibilities [2,20]. Prior research has demonstrated that anticipated caregiving responsibilities meaningfully influence career planning, employment expectations, and work-related decision-making. These findings suggest that hypothetical caregiving scenarios can evoke meaningful cognitive and motivational processes relevant to career-related intentions. Therefore, assessing anticipated caregiving provides theoretically and ecologically meaningful insight into the psychological processes underlying retention intentions, particularly among individuals who have not yet assumed caregiving roles. Future research should include employees who are currently engaged in caregiving to examine whether these findings generalize to real-world caregiving contexts.

Finally, future research should examine whether these patterns generalize across different organizational contexts and cultural settings, particularly given the potential influence of cultural norms regarding caregiving roles, which may shape employees’ perceptions of supervisor support and psychological safety.

Despite these limitations, the study contributes theoretically by identifying psychological safety as a context-sensitive relational resource that bridges professional and caregiving domains. Additionally, psychological safety is frequently conceptualized and measured as a team-level emergent state rather than solely as an individual perception. Although individual-level assessments remain theoretically meaningful—particularly in supervisor–subordinate relationships—future studies would benefit from employing multilevel designs that incorporate both individual and team-level psychological safety. Such approaches would clarify whether the effects observed in the present study persist when psychological safety is treated as a shared climate rather than a personal perception. These future investigations will help clarify the generalizability and robustness of the present findings.

## Supporting information

S1 Table Supplementary tables supporting exploratory factor analysis and correlation results. (DOCX)

S1 Dataset Anonymized raw dataset used for all statistical analyses. (XLSX)

S1 Codebook Variable definitions and coding information corresponding to the anonymized dataset. (DOCX)

S1 File Structural equation model diagram used in the SEM analyses (AMOS). (PDF)
