# Supplementary material for: Psychological safety as a context-sensitive predictor of retention intentions: Gendered effects of supervisor support under caregiving-assumed conditions
Source: PLoS One. 2026 Apr 6;21(4):e0346791. doi: 10.1371/journal.pone.0346791 (PMC13052842; doi:10.1371/journal.pone.0346791)
Supplement: S1 Table — (DOCX) [file pone.0346791.s001.docx]

**S1 Table. Supplementary tables supporting exploratory factor analysis and correlation results.**

**Table A. Results of exploratory factor analysis and reliability for emotional support, instrumental support, and psychological safety (*N* = 522).**

| **Measure** | **Items** | **Extraction  Method** | **Factor Loadings  (Range)** | **Eigenvalue** | **Variance Explained (%)** | ***χ²(df), p, α*** |
| --- | --- | --- | --- | --- | --- | --- |
| Emotional Support | 8 | Maximum Likelihood | .75–.81 | 5.17 | 59.54 | 219.46 (20), < .001, 0.92 |
| Instrumental Support | 6 | Maximum Likelihood | .72–.84 | 4.12 | 62.33 | 238.51 (9), < .001, 0.91 |
| Psychological Safety | 9 | Maximum Likelihood | .73–.83 | 5.47 | 60.79 | 260.32 (27), < .001, 0.93 |

**Note**: Extraction method = Maximum likelihood. All factor loadings are significant at *p* < .001.

**Table B. Correlations among parcels and outcome variables (*N* = 522).**

|  | Variable | *M* | *SD* | 1 | 2 | 3 | 4 | 5 | 6 | 7 | 8 | 9 | 10 | 11 |
| --- | --- | --- | --- | --- | --- | --- | --- | --- | --- | --- | --- | --- | --- | --- |
| 1 | ES1 | 2.99 | 0.99 | — |  |  |  |  |  |  |  |  |  |  |
| 2 | ES2 | 3.22 | 0.97 | .77^***^ | — |  |  |  |  |  |  |  |  |  |
| 3 | ES3 | 3.07 | 0.98 | .78^***^ | .83^***^ | — |  |  |  |  |  |  |  |  |
| 4 | IS1 | 3.23 | 1.05 | .64^***^ | .74^***^ | .66^***^ | — |  |  |  |  |  |  |  |
| 5 | IS2 | 3.17 | 1.05 | .65^***^ | .74^***^ | .67^***^ | .77^***^ | — |  |  |  |  |  |  |
| 6 | IS3 | 2.99 | 1.04 | .58^***^ | .60^***^ | .58^***^ | .60^***^ | .71^***^ | — |  |  |  |  |  |
| 7 | PS1 | 4.32 | 1.30 | .45^***^ | .56^***^ | .49^***^ | .45^***^ | .47^***^ | .33^***^ | — |  |  |  |  |
| 8 | PS2 | 4.16 | 1.25 | .46^***^ | .53^***^ | .52^***^ | .42^***^ | .44^***^ | .35^***^ | .83^***^ | — |  |  |  |
| 9 | PS3 | 4.12 | 1.21 | .54^***^ | .60^***^ | .58^***^ | .51^***^ | .52^***^ | .44^***^ | .79^***^ | .83^***^ | — |  |  |
| 10 | RI-normal | 3.86 | 1.57 | .38^***^ | .41^***^ | .41^***^ | .36^***^ | .41^***^ | .36^***^ | .31^***^ | .33^***^ | .38^***^ | — |  |
| 11 | RI-caregiving | 2.96 | 1.20 | .40^***^ | .41^***^ | .42^***^ | .35^***^ | .38^***^ | .39^***^ | .46^***^ | .52^***^ | .59^***^ | .61^***^ | — |

**Note.** ES = Emotional Support parcels; IS = Instrumental Support parcels; PS = Psychological Safety parcels; RI = Retention intention. Values represent Pearson correlation coefficients. *N* = 522. **p* < .05, ***p* < .01, ****p* < .001.
