# Supplementary material for: Psychological safety as a context-sensitive predictor of retention intentions: Gendered effects of supervisor support under caregiving-assumed conditions
Source: PLoS One. 2026 Apr 6;21(4):e0346791. doi: 10.1371/journal.pone.0346791 (PMC13052842; doi:10.1371/journal.pone.0346791)
Supplement: S1 Codebook — (DOCX) [file pone.0346791.s003.docx]

**Codebook for S2・S3 Dataset**

**Description.** This file provides variable definitions for the anonymized participant-level dataset submitted as Supporting Information (S2・S3 Dataset).

**Response scales.**

Retention intention: values 1–5(5-point Likert scale).

Emotional support items: values 1–5(5-point Likert scale).

Instrumental support items: values 1–5(5-point Likert scale).

Psychological safety items: values 1–7(7-point Likert scale).

**Identifiers**

| Variable | Description | Type | Values / Range |
| --- | --- | --- | --- |
| case_id | Anonymous participant ID (sequential number). | Numeric | 1 to 522 |

**Demographics and design**

| Variable | Description | Type | Values / Range |
| --- | --- | --- | --- |
| Age | Participant age in years. | Numeric | 20 to 39 |
| Gender | Participant gender (coded). | Numeric | Values / Range:  1 = male  2 = female |
| Child | Parental status (coded). | Numeric | Values / Range:  0 = no child  1 = has child |

**Retention intention**

| Variable | Description | Type | Values / Range |
| --- | --- | --- | --- |
| Retain_ care | Retention intention under assumed family-care condition (single-item measure). | Numeric | 1–5 (5-point Likert scale; higher values indicate stronger intention to remain employed) |
| Retain_ general | Retention intention under general condition (single-item measure). | Numeric | 1–5 (5-point Likert scale; higher values indicate stronger intention to remain employed) |

**Emotional support items**

| Variable | Description | Type | Values / Range |
| --- | --- | --- | --- |
| es1m1 | Emotional support item 1. | Numeric | 1–5 (5-point Likert scale) |
| es1m2 | Emotional support item 2. | Numeric | 1–5 (5-point Likert scale) |
| es1m3 | Emotional support item 3. | Numeric | 1–5 (5-point Likert scale) |
| es1m4 | Emotional support item 4. | Numeric | 1–5 (5-point Likert scale) |
| es1m5 | Emotional support item 5. | Numeric | 1–5 (5-point Likert scale) |
| es1m6 | Emotional support item 6. | Numeric | 1–5 (5-point Likert scale) |
| es1m7 | Emotional support item 7. | Numeric | 1–5 (5-point Likert scale) |
| es1m8 | Emotional support item 8. | Numeric | 1–5 (5-point Likert scale) |

**Instrumental support items**

| Variable | Description | Type | Values / Range |
| --- | --- | --- | --- |
| isu1m9 | Instrumental support item 9. | Numeric | 1–5 (5-point Likert scale) |
| isu1m10 | Instrumental support item 10. | Numeric | 1–5 (5-point Likert scale) |
| isu1m11 | Instrumental support item 11. | Numeric | 1–5 (5-point Likert scale) |
| isu1m12 | Instrumental support item 12. | Numeric | 1–5 (5-point Likert scale) |
| isu1m13 | Instrumental support item 13. | Numeric | 1–5 (5-point Likert scale) |
| isu1m14 | Instrumental support item 14. | Numeric | 1–5 (5-point Likert scale) |

**Psychological safety items**

| Variable | Description | Type | Values / Range |
| --- | --- | --- | --- |
| ps3m1 | Psychological safety item 1. | Numeric | 1–7 (7-point Likert scale) |
| ps3m2 | Psychological safety item 2. | Numeric | 1–7 (7-point Likert scale) |
| ps3m3 | Psychological safety item 3. | Numeric | 1–7 (7-point Likert scale) |
| ps3m4 | Psychological safety item 4. | Numeric | 1–7 (7-point Likert scale) |
| ps3m5 | Psychological safety item 5. | Numeric | 1–7 (7-point Likert scale) |
| ps3m6 | Psychological safety item 6. | Numeric | 1–7 (7-point Likert scale) |
| ps3m7 | Psychological safety item 7. | Numeric | 1–7 (7-point Likert scale) |
| ps3m8 | Psychological safety item 8. | Numeric | 1–7 (7-point Likert scale) |
| ps3m9 | Psychological safety item 9. | Numeric | 1–7 (7-point Likert scale) |

**Retention intention items were rated on a 5-point Likert scale:**

1 = strongly disagree

2 = disagree

3 = neutral

4 = agree

5 = strongly agree

**Support items were rated on a 5-point Likert scale:**

1 = strongly disagree

2 = disagree

3 = neutral

4 = agree

5 = strongly agree

**Psychological safety items were rated on a 7-point Likert scale:**

1 = strongly disagree

2 = disagree

3 = somewhat disagree

4 = neutral

5 = somewhat agree

6 = agree

7 = strongly agree

Notes

All data are fully anonymized. The dataset contains no direct personal identifiers (e.g., names, contact information, precise dates, addresses, or institution-specific ID numbers).
