# Supplementary figures and images for: Psychological safety as a context-sensitive predictor of retention intentions: Gendered effects of supervisor support under caregiving-assumed conditions
Source: PLoS One. 2026 Apr 6;21(4):e0346791. doi: 10.1371/journal.pone.0346791 (PMC13052842; doi:10.1371/journal.pone.0346791)

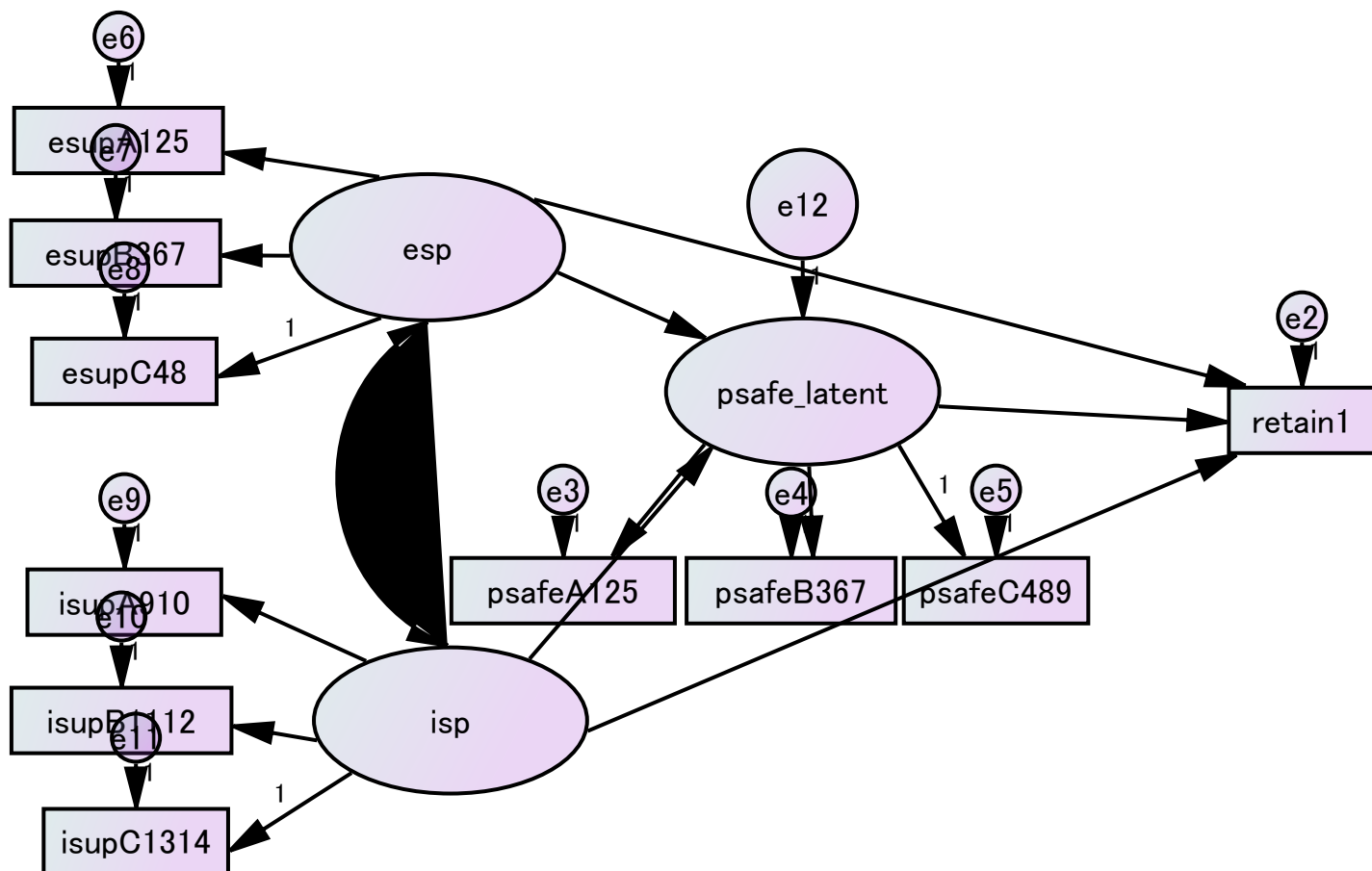

Supplement: S1 File — (PDF) [file pone.0346791.s004.pdf]
